# Supplementary material for: Designing Highly Efficient Cu2O-CuO Heterojunction CO Oxidation Catalysts: The Roles of the Support Type and Cu2O-CuO Interface Effect
Source: Nanomaterials (Basel). 2022 Aug 31;12(17):3020. doi: 10.3390/nano12173020 (PMC9457833; doi:10.3390/nano12173020)
Supplement: Supplementary file 1 [file nanomaterials-12-03020-s001.zip › nanomaterials-1881226-supplementary.pdf]

nanomaterials

Supplementary Material

Designing Highly Efficient Cu_2O-CuO Heterojunction CO Oxidation Catalysts: The Roles of the Support Type and Cu_2O-CuO Interface Effect

Fen Zhao [1,†], Yiyu Shi [1,†], Leilei Xu [1,*], Mindong Chen [1,*], Yingying Xue [1], Cai-E Wu [2], Jian Qiu [3], Ge Cheng [1], Jingxin Xu [4] and Xun Hu [5]

1 Collaborative Innovation Centre of the Atmospheric Environment and Equipment Technology, Jiangsu Key Laboratory of Atmospheric Environment Monitoring and Pollution Control, School of Environmental Science and Engineering, Nanjing University of Information Science & Technology, Nanjing 210044, China
2 College of Light Industry and Food Engineering, Nanjing Forestry University, Nanjing 210037, China
3 Jiangsu Shuangliang Environmental Technology Co., Ltd., Jiangyin 214400, China
4 State Environmental Protection Key Laboratory of Atmospheric Physical Modeling and Pollution Control, China Energy Science and Technology Research Institute Co., Ltd., Nanjing 210023, China
5 School of Material Science and Engineering, University of Jinan, Jinan 250022, China
* Correspondence: leileixu88@gmail.com (L.X.); chenmdnuist@163.com (M.C.)
† These authors contributed equally to this work.

S1. Experimental

S1.1 Support Preparation

S1.1.1 The Synthesis of α-MnO_2 Nanotube Hollow Microsphere Support

The α-MnO_2 nanotube hollow microsphere was prepared by the improved one-pot method according to the previous report [56]. Typically, 0.45 g potassium permanganate (Sinopharm Chemical Reagent Co., Ltd., Shanghai, China) and 1.0 mL concentrated hydrochloric acid (Sinopharm Chemical Reagent Co., Ltd., China) were added to 40 mL deionized water in the sequential manner. After the continuous magnetic stirring for about 20 minutes, the precursor solution was transferred to the 50 mL stainless steel autoclave. Then, the autoclave was placed in the 120 °C oven for 10 h. After the hydrothermal synthesis process, the samples were cooled down to room temperature and was collected by the centrifugation and alternate wash with the anhydrous ethanol and deionized water for several times. Lastly, the prepared brown α-MnO_2 solid powder was dried in the vacuum oven at 65 °C for 6 h.

S1.1.2 The Preparation of CeO_2 Mesoporous Nanosphere

The CeO_2 nanosphere support was prepared by the improved one-pot method according to previous report [57]. In a typical procedure, 1.0 g Ce $(NO_3)_3{\cdot}6H_2O$ (Shanghai Macklin Bio-Chem Co., Ltd., Shanghai, China) was dissolved in 1 mL deionized water. Then, 1 mL acetic acid (Shanghai Meryer Co., Ltd., Shanghai, China) and 30 mL ethylene glycol (Sinopharm Chemical Reagent Co., Ltd., China) were sequentially added under the vigorous agitation. The mixed solution was transferred into the 50 mL stainless steel autoclave and experienced the hydrothermal process at 180 °C for 3 h. After cooling the sample to normal temperature, the product was collected by the centrifugation and alternate wash with the anhydrous ethanol and deionized water. Finally, the prepared sample powder was dried in the vacuum oven at 65 °C for 6 h.

S1.1.3 The Synthesis of Nano ZSM-5 Zeolite

The nano ZSM-5 zeolite was prepared by using the modified one-pot method according to the previous literature report [58]. Typically, 0.1 g NaOH (Shanghai Macklin Bio-

Chem Co., Ltd., China), 15.57 mL tetrapropylammonium hydroxide (TPAOH, 25 wt.%, Shanghai Aladdin Bio-Chem Co., Ltd., China) solution, 2.0 g urea (Shanghai Aladdin Bio-Chem Co., Ltd., Shanghai, China), 0.12 mL iso-Propyl alcohol (IPA, Shanghai Aladdin Bio-Chem Co., Ltd., China) and 11.9 mL tetraethyl orthosilicate (TEOS, Shanghai Aladdin Bio-Chem Co., Ltd., China) were mixed with 15.7 mL of ultrapure water under vigorous stirring so that it was completely dissolved. Then, the resultant solution was carried out the hydrothermal synthesis process in a rotating oven at 180 °C for 48 h with 30 rpm. After the hydrothermal synthesis process, the solids were separated by centrifugation and alternate wash with absolute ethanol and ultrapure water for 6 times. Finally, the separated solids were dried in a 65 °C oven and then calcined at 550 °C for 6 h with the heating rate of 2 °C/min.

S1.2 The Preparation of the Catalysts

S1.2.1 The Preparation of Cu_2O/S Supported Catalysts

A series of the Cu_2O/S supported catalyst was prepared by the liquid phase reduction deposition precipitation synthesis strategy, where the "S" stands for different supports (S = α-MnO_2, CeO_2, ZSM-5, and Fe_2O_3 (Sinopharm Chemical Reagent Co., Ltd., China)). The Cu_2O loading amount was fixed at 15%. For the specific procedure, the 0.283 g support was ultrasonic dispersed into 100 mL deionized water, and then 0.25 mmol (0.05 g) copper acetate (Shanghai Macklin Bio-Chem Co., Ltd., China) and 0.005 mmol (0.29 g) polyvinylpyrrolidone (Shanghai Aladdin Bio-Chem Co., Ltd., China) were dissolved in the above solution with the support. Besides, 5mmol (0.2 g) NaOH was dissolved in 20 mL deionized water. Subsequently, the prepared NaOH solution (0.25 mol/L) was gradually dropped into the mixed solution containing the copper acetate precursor and the support at room temperature together with vigorous agitation. After that, the blue suspended substance in the solution was observed. Meanwhile, 0.75 mmol (0.132 g) ascorbic acid (Shanghai Aladdin Bio-Chem Co., Ltd., China) was dissolved in 15 mL deionized water to form a transparent ascorbic acid solution (0.05 mol/L). Then, the obtained 0.05 mol/L ascorbic acid aqueous solution was added into the above blue suspension with the rate of the 3 drops/s and vigorous agitation. The color of the suspension solution experienced blue, green, light orange, and brick red colors with time. The product was obtained after the alternate centrifugation and wash for four times. Finally, the as-prepared Cu_2O/S catalyst powder was dried in the vacuum oven at 55 °C for 6 h.

S1.2.2 The Preparation of Cu_2O-CuO Heterojunction Catalysts

The Cu_2O-CuO/α-MnO_2 heterojunction catalysts with various Cu^+/Cu^{2+} ratios were synthesized by calcining the Cu_2O/α-MnO_2 at 240, 260 and 280 °C, respectively. Concretely, the Cu_2O/α-MnO_2 was calcined at the targeted calcination temperature for 1 h with the heating rate of 0.5 °C/min. The obtained Cu_2O-CuO/α-MnO_2 heterojunction catalyst was named as the Cu_2O-CuO/α-MnO_2-T, in which "T" represented the targeted calcination temperature. Likewise, CuO/α-MnO_2 catalyst, named as the Cu_2O/α-MnO_2-500, was prepared by calcining at 500 °C for 1 h.

S2. Catalyst Characterizations

The patterns of the X-ray powder diffraction (XRD) were measured and recorded on the XD-6100 X-ray diffractometer (Shimazu, Japan) with the Cu K radiation (40kV, 40 mA) and scanning speed 10°/min in the range of 10-80° (2θ).

The thermogravimetric analysis (TGA) was analyzed On the Thermo Plus EVO TG/DTA device (Thermo Fisher Scientific, USA). The sample was heated under the air atmosphere in the range of 30 °C to 900 °C with the heating rate of 10 °C/min.

The physical adsorption of N_2 was measured on the Autosorb-IQ-Ag-MP device (Quantachrome, USA) at liquid nitrogen temperature (−196 °C). The sample was degassed at 200 °C for 3 h before the normal analysis to eliminate the surface-adsorbed water and

contaminants. The multipoint Brunauer-Emmett-Teller (BET) method was used to calculate the specific surface area of the catalyst in the range of 0–1.0 P/P_0, and the Barrett-Joyner-Halenda (BJH) method was used to calculate the pore size distribution and pore volume from the adsorption branch of the isotherm.

The Scanning electron microscopy (SEM) photos were collected on the Apreo S Hivac equipment (Thermo Fisher Scientific, USA) with the acceleration voltage of 5 kV.

The transmission electron microscopy (TEM) Images were recorded on the TECNAI G2 F20 transmission electron microscope (FEI, USA) with the 200 kV acceleration voltage. The sample was ultrasonically dispersed in anhydrous ethanol for 30 min, and afterwards dropped on the carbon-coated copper grid.

The Fourier transformed infrared spectroscopy (FTIR) was obtained by using the Nicolet iS5 (Thermo Fisher Scientific, USA) as the spectrometric analysis tool.

The X-ray photoelectron spectroscopy (XPS) measurements was performed on the Thermo Scientific K-Alpha+ spectrometer (Thermo Fisher Scientific, USA). The binding energies of the XPS spectra were adjusted and calibrated by using the C 1s binding energy of 284.5eV as the reference. The penetration depth of each catalyst was approximately 1–2 nm.

The H_2 temperature-programmed reduction (H_2-TPR) was determined on the self-made fixed bed reactor, and the consumption curve of H_2 was recorded by using the online LCD200 mass spectrometry (TILON, USA). For each test, 5 mg of the catalyst was loaded and the mixture of the H_2 and Ar (flow rates are 0.4 mL/min and 7.6 mL/min, respectively) was used as the reducing agent. The H_2-TPR was conducted from room temperature to 800 °C at the heating rate of 20 °C /min after the H_2 signal baseline (m/z = 2) was steady.

S3. Catalytic Activity Measurements

The catalytic property of catalysts for CO oxidation was tested on the perpendicular fixed bed continuous flow reactor equipped with quartz tube (I.D. = 8.00 mm). The mass flow controllers (MFC) were used to regulate the flow of the gases and two thermocouples located in the center of the fixed-bed were used to precisely monitor and control the temperature of the reaction.

In a typical experiment, 100mg catalyst was used in each test and the inlet gas containing 1% CO, 20% O_2, and 79% N_2 were used as the reaction gas. The total flow rate was 20 mL/min, with a gas space velocity of 12,000 mL/(g·h). The catalytic activity of CO oxidation on several catalysts was evaluated in the given temperature range. Besides, the 12-hour stability test was performed at 90 °C with a GHSV of 12000 mL/(g·h). Finally, the output gas was online analyzed on the Techcomp GC-7900 gas chromatograph equipped with the thermal conductivity detector (TCD). CO conversion reflected the catalytic activity of the catalyst. In addition, the CO conversion could be calculated based on the carbon balance. The $F_{CO,\, inlet}$ and $F_{CO,\, outlet}$ in the below formula indicated the inlet and outlet flows of CO in the reactor, respectively

$$C_{CO} = \frac{F_{CO,\, inlet} - F_{CO,\, outlet}}{F_{CO,\, inlet}} \times 100\% \quad (1)$$

S4. Figures

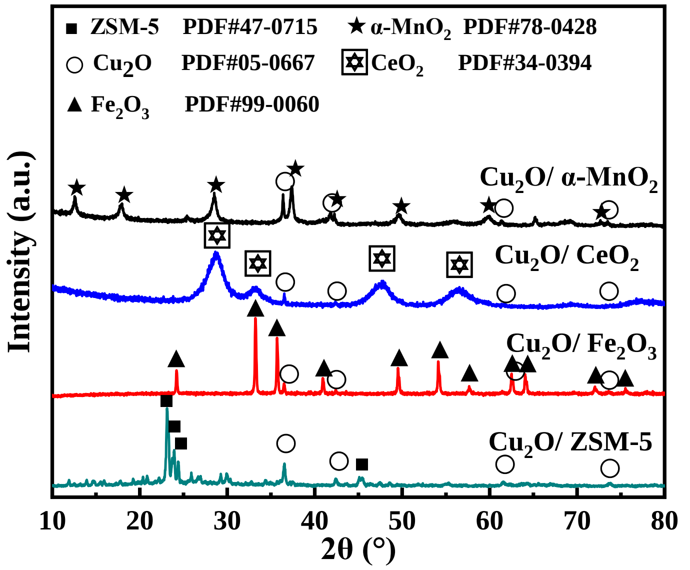

Figure S1. X-ray diffraction patterns of the as-prepared Cu_2O/S (S = ZSM-5, CeO_2, α-MnO_2 and commercial Fe_2O_3) catalysts.

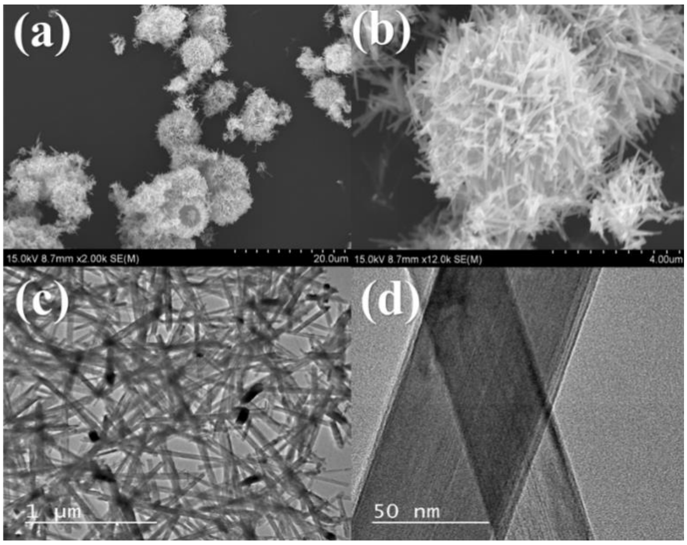

Figure S2. SEM (**a**,**b**) and TEM (**c**,**d**) image of the as-prepared α-MnO_2 supports.

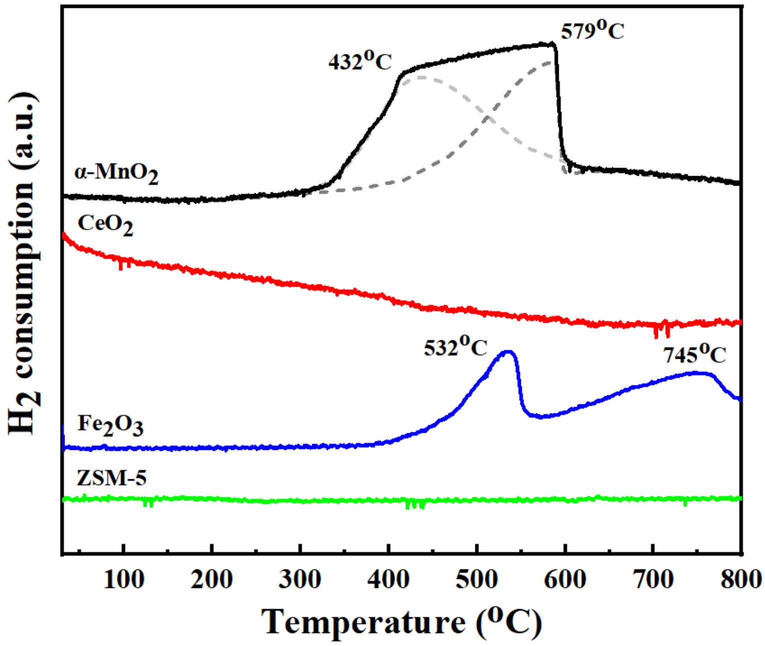

Figure S3. H_2-TPR profiles of the as-prepared ZSM-5, CeO_2, α-MnO_2 and Fe_2O_3 supports.

References

56. Chen, R.; Yu, J.; Xiao, W. Hierarchically porous MnO_2 microspheres with enhanced adsorption performance. *J. Mater. Chem. A*. **2013**, *1*, 11682–11690. https://doi.org/10.1039/C3TA12589K.
57. Wu, K.; Zhou, L.; Jia, C.-J.; Sun, L.-D.; Yan, C.-H. Pt-embedded-CeO_2 hollow spheres for enhancing CO oxidation performance. *Mater. Chem. Front.* **2017**, *1*, 1754–1763. https://doi.org/10.1039/C7QM00244K
58. Li, T.; Krumeich, F.; Ihli, J.; Ma, Z.; Ishikawa, T; Pinar, A.B.; van Bokhoven, J.A. Heavy atom labeling enables silanol defect visualization in silicalite-1 crystals. *ChemComm*. **2019**, *55*, 482–485. https://doi.org/10.1039/C8CC07912A.
